# Analysis of Post-Bonding Crack-Induced Double Cantilever Bending (PDC-DCB) Method for Hybrid Bonding Strength Measurement

**DOI:** 10.3390/ma18245480

**Published:** 2025-12-05

**Authors:** Cong Mei, Tianze Zheng, Qiuhan Hu, Yingjie Chen, Yuan Xu, Huiyao Zhao, Liu Chang, Yuan Yuan, Zongguang Yu, Liyi Li

**Affiliations:** 1 School of Integrated Circuits, Southeast University, Wuxi 214000, China; 230229587@seu.edu.cn (C.M.); 220236463@seu.edu.cn (T.Z.); 220205825@seu.edu.cn (Y.C.); 220226140@seu.edu.cn (Y.X.); 220216003@seu.edu.cn (H.Z.); 230239483@seu.edu.cn (L.C.); 2 Technology Development & Central Laboratory, Shenzhen Kaifa Technology Co., Ltd., Shenzhen 518000, China; 3 School of Advanced Technology, Xi’an Jiaotong-Liverpool University, Suzhou 215123, China; qiuhan.hu23@student.xjtlu.edu.cn; 4 The 58th Research Institute of China Electronics Technology Group Corporation, Wuxi 214000, China; 15629070655@163.com (Y.Y.); yuzg58@163.com (Z.Y.)

**Keywords:** hybrid bonding (HB), bonding strength, double cantilever bending (DCB) test, finite element analysis (FEA)

## Abstract

The quantitative measurement of bonding strength in hybrid bonding (HB) is an indispensable metrology for process and reliability evaluation. Methods currently used such as blade insertion (BI) and double cantilever bending (DCB) have suffered from a wafer edge-only capability and limited repeatability due to a lack of precise interface crack initiation. This study reports an improved DCB method by introducing a post-bonding crack (PBC) to avoid undesired cracking in a wafer substrate during DCB propagation. This method is firstly applied to measure the bonding strength of SiCN-SiCN under O_2_ and N_2_ activation. The test data shows that the bonding strengths are 3.53 J/m^2^ and 2.93 J/m^2^ with the deviations less than 3.84% and 1.84%. Based on the experimental data, finite element analysis (FEA) methods are used to simulate the crack propagation process of the PBC-DCB method. The results show that the bonding interface crack propagation can be accurately described by an optimized viscoelastic exponential model. The accuracy of simulation increased from 16.06% to 1.77%. Finally, it was found that the PBC-DCB method can solve the issue in conventional DCB where the crack may be initiated away from the target interface, therefore measuring the wrong interface. This advantage is further validated by simulations considering the offset of the PBC away from the bonding interface.

## 1. Introduction

In recent years, hybrid bonding (HB) has emerged as a pivotal technology in 3D integration to meet the increasing demand for computing power from cloud computing and AI. HB can provide a higher interconnect density, lower power consumption, lower signal delay, and better thermal dissipation. Die-to-wafer (D2W) and wafer-to-wafer (W2W) are two major formats of hybrid bonding [1,2,3]. Through material and process optimization, significant achievements have been made in addressing the reliability issues of hybrid bonding, such as interface voids, thermal stability, and electromigration [4,5]. Studies have shown that an insufficient bonding strength is one of the major causes for interfacial delamination and electrical open failures. Therefore, bonding strength is a key metric for HB process development and reliability evaluation in both research and high-volume production [6].

**Figure 1:**
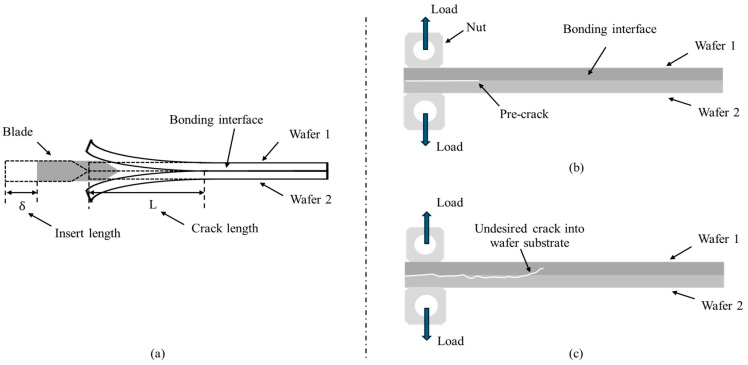
BI and DCB testing methods for HB strength. (**a**) BI method. Crack length measurement is very critical. (**b**) Conventional DCB method. A pre-crack should be fabricated by surface etching before bonding. (**c**) Pre-crack challenge. The crack propagation can lead to undesired cracking into the wafer substrate.

In the community of HB, bonding strength is typically defined as the energy required to break the bonding interface per unit area, in the unit J/m^2^. There are several methods that have been used to measure strength. The indentation method can be used for a rapid comparison of the bonding strength for the same samples, but it is incapable of providing accurate values [7,8]. The blade insertion (BI) technique can measure bonding strength based on crack length, but it is difficult for existing techniques such as infrared cameras to accurately capture the crack length when there is an irregular crack front shape. This issue will become much more challenging with an increased interface interconnect density [9]. Also, the BI method is limited to wafer edge measurements relying on an edge bevel geometry (Figure 1a).

Double cantilever bending (DCB) is a theoretical method that can accurately obtain bonding strength without measuring the crack length for any location within a wafer. In the scheme, a so called “pre-crack” was fabricated by surface etching before bonding and used as the initiation position for DCB (Figure 1b) [10]. However, the major challenge for the conventional DCB method is the lack of precise initiation at the bonding interface. This pre-crack is not practical in most HB cases since it would severely deteriorate the quality of the HB process itself because contamination or excessive surface roughness is introduced during pre-crack generation, which often leads to crack propagation away from the crack and thus affects the measurement of facture energy inside the silicon substrate (Figure 1c).

To overcome this issue, this research first proposes a PBC-DCB method (a novel “post-bonding crack” induced in the DCB method) to solve the problem of precise crack initiation. Then this method is applied to test the interfacial bonding strength of SiCN-SiCN activated by O_2_ or N_2_. Referring to the PBC-DCB testing data, two FEA fracture models (VCCT and CZM) are established for the HB interface crack propagation simulation. Considering that the PBC center may have some offset from the bonding interface, the effective FEM model is applied to simulate this actual issue.

## 2. PBC-DCB Testing Method

### 2.1. PBC-DCB Specimen Preparation

**Figure 2:**
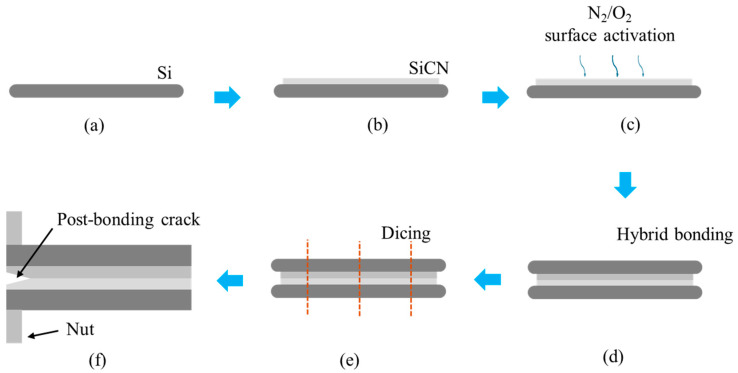
Hybrid bonding process workflow for PBC-DCB test specimen preparation. (**a**) Bare Si cleaning; (**b**) SiCN deposition; (**c**) surface planarization and activation; (**d**) bonding; (**e**) specimen dicing; (**f**) post-bonding crack fabrication and nuts attachment.

A wafer-to-wafer hybrid bonding process workflow is followed to prepare the specimen for PBC-DCB tests. As shown in Figure 2, clean 12-inch bare silicon (Si) wafers are used as substrates. A layer of silicon carbon nitride (SiCN) film is deposited on the top surface of the Si substrate using plasma-enhanced chemical vapor deposition (PECVD). Then the SiCN surface is planarized using chemical mechanical polishing (CMP) and activated by plasma using N_2_ or O_2_ gas. After activation, two wafers are bonded face-to-face using fully automated hybrid bonding equipment. The bonded wafers are diced into 40 × 5 mm^2^ test strips. Finally, nuts are attached at the front of specimen using glue at room temperature.

A laser is used to etch the edge-bonded interface to form a post-bending crack after HB. The cutting laser has a frequency of 20 kHz, a pulse width of 40 μs, a processing count of 100 times, and a moving speed of 10 mm/s. The beam can be precisely aligned to the interface using a close-loop wafer stage with a microscope. The final offset between the post-bonding crack and the bonding interface is thus controlled within 5 μm.

**Figure 3:**
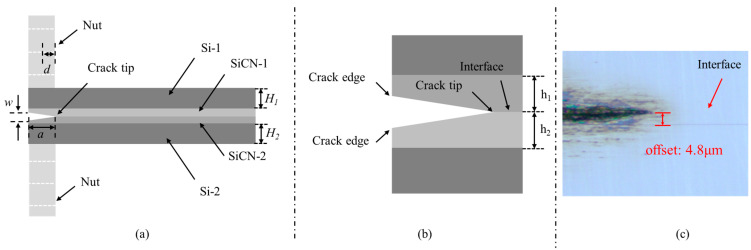
PBC-DCB testing method structure. (**a**) Side view of PBC-DCB structure, where the post-bending crack (PBC) is a trigonal prism; (**b**) magnification of the crack tip located at the bonding interface; (**c**) the crack tip offset value between the post-bonding crack and bonding interface.

The configuration of the PBC-DCB test samples is shown in Figure 3. The thickness of the silicon substrates and SiCN films are H_i_ and h_i_, respectively. The index *i* = 1, 2 indicates the top and bottom wafers, respectively.

After preparation, the specimens are loaded onto a tensile tester for DCB testing. The load force is applied through the two nuts and load–displacement data is recorded.

### 2.2. PBC-DCB Testing

Based on cantilever beam theory and assuming a plane-strain condition [11] and that the beams are the same thickness (*H*_1_ = *H*_2_ = *H*, *h*_1_ = *h*_2_ = *h*, the bonding strength in the PBC-DCB method is calculated using equations as follows:

(1) $$C=\frac{1}{E_{s}I_{2}{\lambda_{c}}^{3}}\left(\frac{{\lambda_{c}}^{3}\left(2a^{3}-2d^{3}\right)}{3}+2{a^{2}\lambda }_{c}^{2}+2ad\left(\lambda_{c}^{2}+\lambda_{c}\right)+d\lambda_{c}+1\right)+\frac{3\left(a-d\right)}{bG_{s}H}$$

(2) $$G=\frac{P^{2}}{2E_{s}I_{2}b{\lambda_{c}}^{3}}\left({{2a^{2}\lambda }_{c}}^{3}+4a\lambda_{c}^{2}+2d\left(\lambda_{c}^{2}+\lambda_{c}\right)\right)+\frac{3P^{2}}{2b^{2}G_{s}H}$$

where

- *C* is the compliance;
- *G* is the bonding energy;
- *E*_s_ is the Young’s modulus of Si;
- *E*_c_ is the Young’s modulus of SiCN;
- $v_{s}$ is Poisson’s ratio of Si;
- $v_{c}$ is Poisson’s ratio of SiCN;
- $I_{2}$ is the moment of inertia;
- a is the post-bending crack propagation length during DCB testing;
- d is the half-length of nut;
- $G_{s}$ is the shear modulus of Si;
- *b* is the width of Si and SiCN;
- *H* is the thickness of Si;
- *h* is the thickness of SiCN;
- *G* is the bonding strength of SiCN-SiCN interface;
- *P* is the load of beam;
- $\lambda_{c}$ is a function of the modulus and moment of inertia.

$$\lambda_{c}^{4}=\frac{3E_{c}}{\left(1-v_{c}^{2}\right)E_{s}hH^{3}}$$

From the above expression, with the load *P* and measured displacement *δ* during testing, the compliance *C*, crack length a, and bonding strength Gc can be determined by G. The values of the parameters used in the calculation are shown in Table 1.

During PBC-DCB testing, the nut is stretched with a constant displacement velocity of 0.01 mm/min in an atmosphere of pure N_2_ (moisture level < 1 ppm). The tensile tester has a load cell with a range of 0–200 N and an accuracy of ±0.02% of the full scale. The displacement measurement accuracy is ±0.5% of the displayed value. As displacement load grows with a constant rate at blocks, the loading force is recorded by the tester. When noticing a drop in loading force, which means the crack has been propagated, the load is withdrawn so that beams can return to their initial position. These load–unload cycles are repeated until the specimen interface is completely separated.

**Figure 4:**
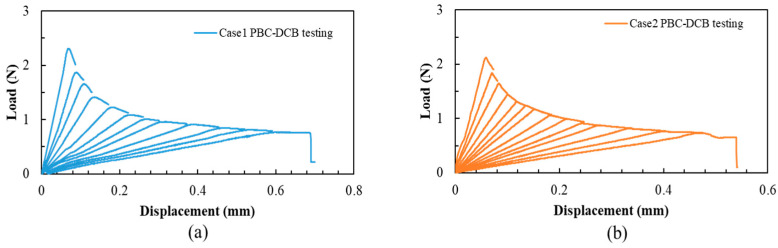
Load–displacement curves obtained from PBC-DCB testing. (**a**) L-D curve for Case 1; (**b**) L-D curve for Case 2.

The specimens activated in O_2_ and N_2_ gas are marked as Case 1 and Case 2. The load–displacement (L-D) curves of Case 1 and Case 2 specimens are displayed in Figure 4.

**Figure 5:**
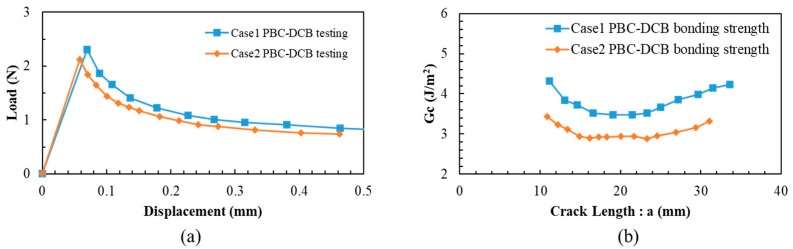
The bonding strength calculation of PBC-DCB testing L-D curve. (**a**) Peak value of L-D curve for Case 1 and Case 2. (**b**) Bonding strength calculation for Case 1 and Case 2.

From Equations (1) and (2), the bonding strength G_c_ and crack length can be obtained from the peak values of the L-D curves. The results are shown in Figure 5.

### 2.3. Bonding Strength Gc–Crack Length a Curves Analysis

**Figure 6:**
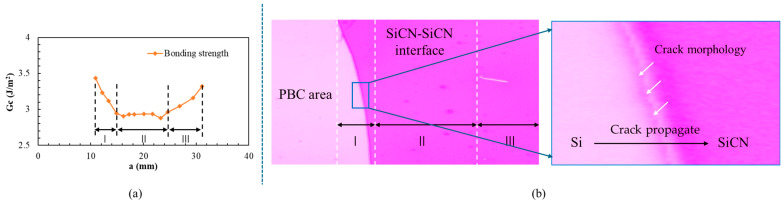
(**a**) Plot of bonding strength *G*_c_ vs. crack length *a*. (**b**) Microscope inspection of the crack interface after PBC-DCB test.

The calculated results indicate that bonding strength varies with crack length a. The Gc-a curves can be divided into three stages, as defined in Figure 6:

- Bonding strength decreases with crack propagation.
- Bonding strength is stable with crack propagation.
- Bonding strength increases with crack propagation.

The crack interface after the PBC-DCB tests is inspected under a microscope to help explain the difference among the three stages. The results indicate that in the first stage, part of the initial crack is located at the Si-SiCN interface rather than at the bonding interface (Figure 6b). Therefore, a higher strength is required during crack propagation, leading to a larger bonding strength of strength calculated in the test. When the crack propagates by approximately 15 mm, the Gc vs. a becomes constant. At this point, it can be considered that the crack is propagating stably along the interface. In the third stage, as the end of the sample is not fixed, the cantilever beam may experience rotation, which results in the test results including opening (mode I) and tearing (mode III) cracks. Thus, the bonding strength once again becomes larger than the expected value.

**Figure 7:**
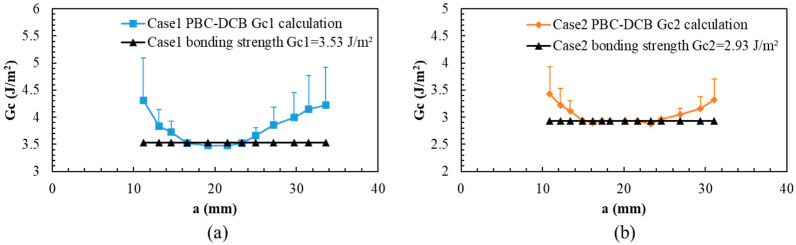
PBC-DCB bonding strength of SiCN-SiCN under O_2_ and N_2_ activation. (**a**) Case 1: specimens activated in O_2_ gas. (**b**) Case 2: specimens activated in N_2_ gas.

Therefore, it is suggested that the bonding strength of region II should be treated as the bonding strength of the SiCN-SiCN interface. The value is shown in Figure 7.

**Figure 8:**
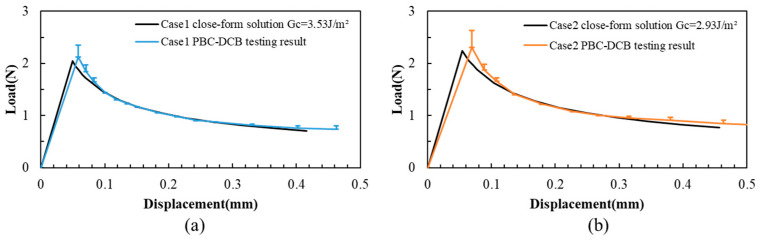
L-D of PBC-DCB testing and closed-form solutions. (**a**) Case 1: specimens activated in O_2_ gas. (**b**) Case 2: specimens activated in N_2_ gas.

To validate the assumption, the load–displacement curves of closed-form solutions are also calculated using Equations (1) and (2). As shown in Figure 8, part of the testing load–displacement curve in region II overlaps with the closed-form results (deviation: 0.4%), while there are some offsets in region I (deviation: 6.3%) and region III (deviation: 3.4%).

## 3. Finite Element Simulation Model

The fracture behavior at the bonding interface can be further understood using a finite element simulation of the above load–displacement curves. At present, various finite element models to describe the mechanics of crack propagation have been developed. Among them, two major models are the virtual crack closure technique (VCCT) and the cohesion model method (CZM) [12,13].

### 3.1. Virtual Crack Closure Technology (VCCT) Model

The virtual crack closure technique (VCCT) has been widely applied in crack propagation or delamination simulations based on linear elasticity fracture mechanics [14].

The VCCT method has the following assumptions:

(1) The crack must propagate along a defined path or interface.
(2) The crack must grow at the interface of linear elastic materials.

In this part, the linear fracture criterion is expressed below:

(3) $$f=\frac{G_{c}}{G_{T}}=\frac{G_{c}}{G_{TI}+G_{TII}+G_{TIII}}$$

where G_TI_, G_TII_, and G_TIII_ refer to the fracture energy of mode I, II, and III. When only a mode I crack is considered, the equation can simplify to

(4) $$f=\frac{G_{c}}{G_{T}}=\frac{G_{c}}{G_{TI}}$$

**Figure 9:**
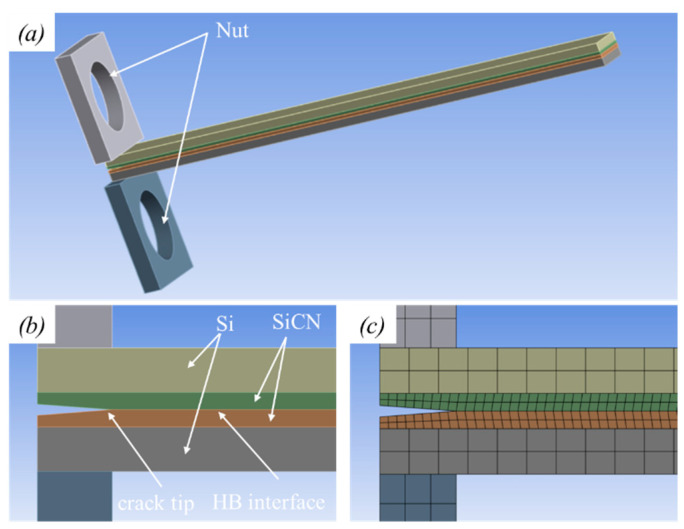
Geometry of PBC-DCB SiCN-SiCN test structure. (**a**) Schematic for FEA HB structure; (**b**) magnification of PBC, where the crack tip is located at bonding interface; (**c**) mesh of PBC structure.

From the PBC-DCB testing results, the Gc of SiCN-SiCN with N_2_ activation is 2.93 J/m^2^. Thus, the fracture criterion for VCCT simulation is set to G_T_ = 2.93 J/m^2^. A finite element geometric SiCN-SiCN hybrid bonding structure model is established, as shown in Figure 9. A mesh size of 0.15 mm is used for the SiCN layer.

**Figure 10:**
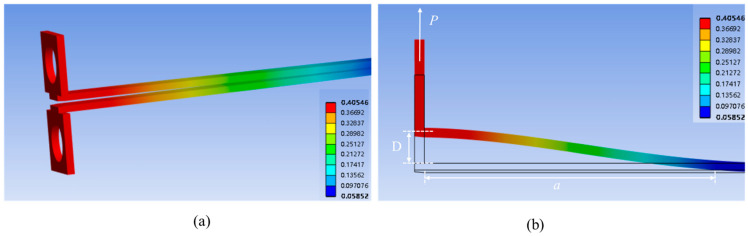
Total deformation of the VCCT model of PBC-DCB testing result. (**a**) Total deformation inspection of the structure; (**b**) the displacement of unilateral nuts and the crack length.

In the model, the crack propagation path is constricted to the SiCN-SiCN interface. The nuts have a displacement of 0.4 mm. A snapshot of the beam deformation in the simulation is shown in Figure 10. The crack length and load force can also be obtained.

**Figure 11:**
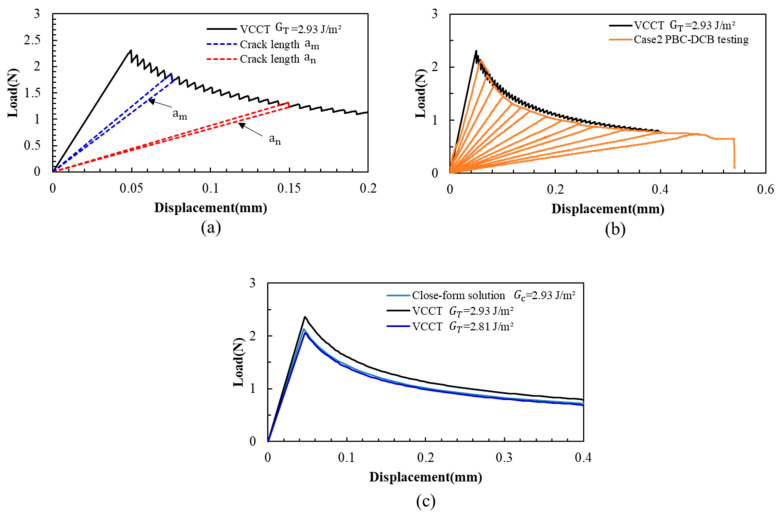
Load–displacement curves of VCCT PBC-DCB model. (**a**) VCCT crack propagation process. The load increase and decrease are correlated with the crack length. (**b**) The L-D curve of VCCT has a gap with PBC-DCB. (**c**) The peak value of load–displacement curves: black curve: the VCCT simulation with input fracture energy *G_T_* = 2.93 J/m^2^; dark blue: the VCCT simulation with input fracture energy *G_T_* = 2.81 J/m^2^; light blue curve: closed-form solution with *G_c_* = 2.93 J/m^2^.

The VCCT L-D is shown in Figure 11. It can be found that the L-D is a typical DCB crack propagation process: as the displacement at the loading point increases at a constant rate, the peak load is strongly correlated with the crack length, and the peak load gradually decreases with intermittent crack jumps.

The VCCT-simulated result of the L-D curve has a significant discrepancy (Figure 11c). However, if the *G_T_* is changed from 2.93 J/m^2^ to 2.81 J/m^2^, the load–displacement curve closely matches the experimental results.

This indicates that the VCCT fracture model cannot be used to simulate this PBC-DCB testing process. This discrepancy may be caused by the following reason: the VCCT model is only suitable for the analysis of the crack propagation or delamination between linear elastic materials, whereas the multilayer composite structure of SiCN-SiCN or the interface in the experiment may exhibit viscoelastic deformation during cantilever beam tension and separation.

### 3.2. Cohesive Zone Model (CZM)

The Cohesive Zone Model (CZM) is another FEA model that can be used to simulate interfacial fracture behavior. Furthermore, the CZM is particularly suitable for viscoelastic materials [15].

In the CZM, crack growth is considered to represent the progressive degradation of the material along the direction of crack tip propagation. This degradation process is characterized by the relationship between the interaction (traction) at the cohesive interface and separation under external load. This part will discuss two CZM models:

(1) Bilinear model of interface delamination.
(2) Exponential model of interface delamination.

#### 3.2.1. Bilinear Model for Interfacial Delamination

**Figure 12:**
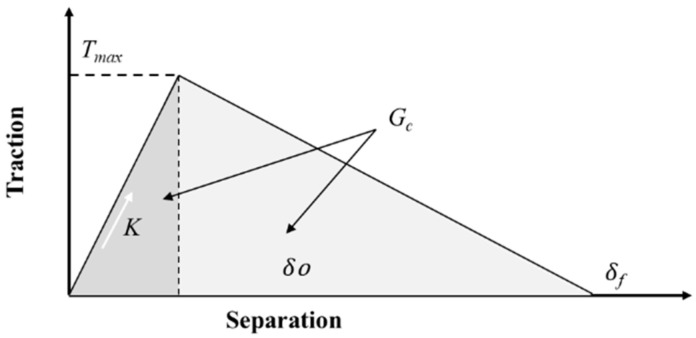
Relationship between normal attraction and interfacial separation for the bilinear CZM model.

For the bilinear model of interface delamination of the CZM [16], the relationship between the force (traction) and the separation distance δ follows the pattern shown in Figure 12.

When the separation is 0~*δ_o_*, the normal traction force increases linearly (the slope reflects the material’s elasticity) until it reaches the maximum value. After reaching the maximum normal traction force *T_max_*, the traction force begins to decrease linearly. The work done by the normal traction force over the whole tensile displacement (corresponding to the area *G_c_*) equals the interfacial bonding strength.

When only mode I crack propagation is considered, the bilinear CZM model has the following expression:

(5) $$G_{c}=\frac{\delta_{f}*T_{max}}{2}$$

(6) $$K=\frac{T_{max}}{\delta_{o}}$$

where

- *Gc* is the interface bonding strength;
- *T_max_* is the maximum normal traction force;
- *δ*_0_ is the normal displacement at the maximum normal traction force;
- *δ_f_* is the normal displacement when crack propagation is completed.

**Figure 13:**
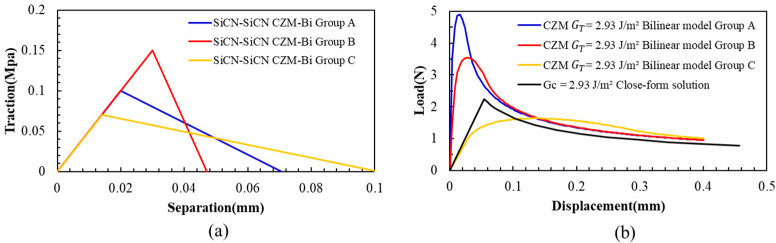
(**a**) Three groups of assumed parameters for the bilinear CZM PBC-DCB model. (**b**) Load–displacement curves for the exponential for interface delamination.

For the PBC-DCB structure, we assume a composite structure of SiCN-SiCN with G_c_ = 2.93 J/m^2^. The crack propagation process of the bilinear CZM model can be simulated by adjusting *δ_f_*, *δ*_0_, and *T_max_*. The correct model parameters can be obtained by comparing the resulting load–displacement curve with the experimental data. Three typical groups of assumed parameters for the bilinear model are listed as group A, group B, and group C in Table 2 and Figure 13.

For the bilinear traction separation curve illustrated in Figure 13a, the area under each set of curves is 2.93 J/m^2^.

Putting these three groups of parameters into the CZM structure for simulation, the load–displacement curves can be obtained. Compared with the experimental results, the simulation results also show a significant discrepancy for all three curves (Figure 13b). This discrepancy indicates that the bilinear model may also not be suitable for this multilayer composite structure of SiCN-SiCN.

The model assumes both the load and displacement change linearly before and after crack propagation. This assumption might be the key reason for the inconsistency with the actual testing situation.

#### 3.2.2. Exponential Model of Interface Delamination

**Figure 14:**
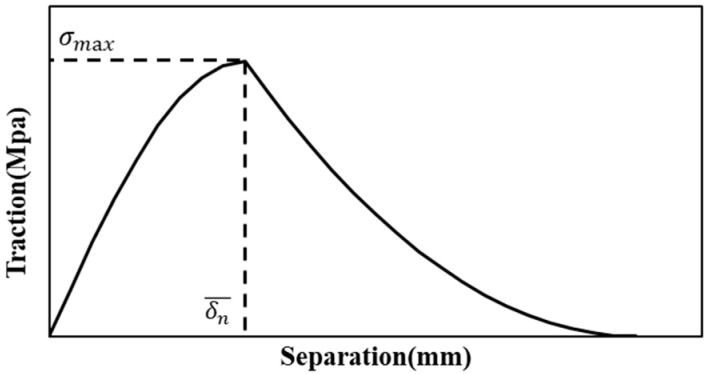
The exponential behavior for exponential CZM model.

The exponential model [4] uses the load and displacement expressed in Figure 14.

The interfacial bonding strength can be expressed as

(7) $$\emptyset \left(\delta \right)=\sigma_{max}\bar{\delta_{n}}\left[1-\left(1+{∆}_{n}\right)e^{-{∆}_{n}}e^{-{∆}_{t}^{2}}\right]$$

When only a mode I crack is considered (no tangential slip crack or stress), the equation simplifies to

(8) $$\emptyset_{n}=m\sigma_{max}\bar{\delta_{n}}$$

where

- $\emptyset_{n}$ is the interfacial bonding strength of a mode I crack,
- $\sigma_{max}$ is the maximum normal traction;
- $\bar{\delta_{n}}\;$ is the normal separation across the interface;
- *m* is an equivalence factor of the exponential model.

Typically, constant m is taken as the base of natural logarithm *e* = 2.71 where the CZM original exponential model has been used. Three groups of parameters, A, B, and C, are listed in Table 3.

**Figure 15:**
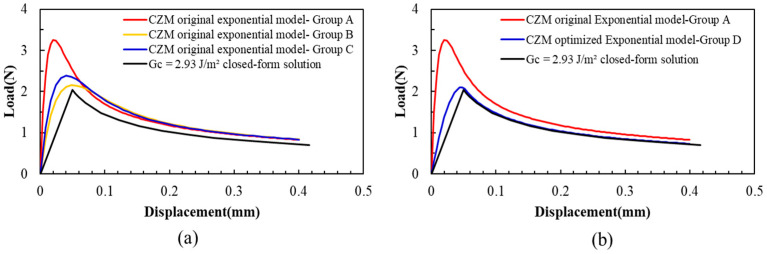
Load–displacement curves for the exponential model for interface delamination. (**a**) The original exponential model, m = e. (**b**) The optimized exponential model m = 3.91.

Substituting these parameters into the model and solving it, the load–displacement curves are shown in Figure 15a. All these simulation results are also different from the closed-form solution result. It can be found that the bonding strength calculated from these curves is lower than the closed-form solution.

However, setting m as 3.91, as shown in Table 4, the load–displacement curve from the simulation matches well with the experimental curves, as shown in Figure 15b.

Compared with the closed-form solution results, when the optimized exponential interface delamination model is adopted and m is 3.91, the FEA can accurately simulate the DCB testing process, as shown in Figure 15b.

**Figure 16:**
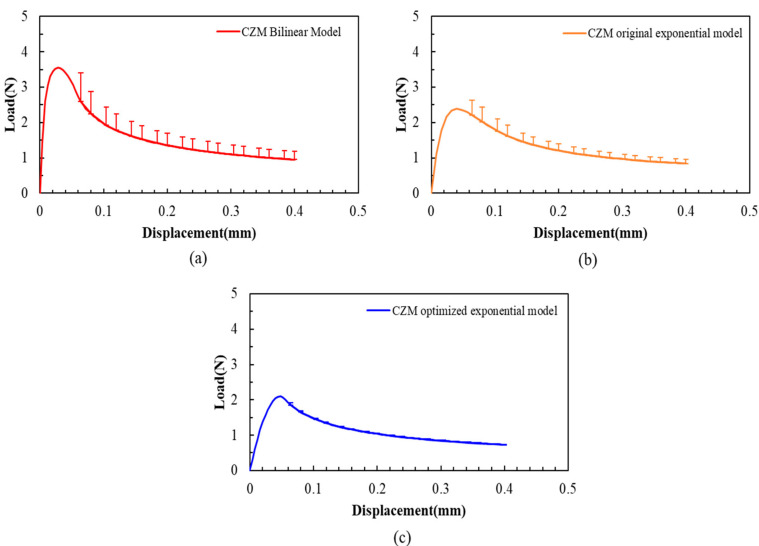
Load–displacement curves and error analysis for different FEA models. (**a**) The CZM bilinear model results; (**b**) the CZM original exponential model; (**c**) the CZM optimized exponential model.

For the CZM bilinear model, the CZM original exponential model, and CZM optimized exponential model, an error bar of L-D curve analysis has been completed for these three FEA models, as shown in Figure 16. The result reveals that the CZM bilinear model has the largest error bar, while the CZM optimized exponential has the smallest error bar.

So far, the most effective FEA model based on the exponential CZM (CZM optimized exponential model) has been obtained to describe the PBC-DCB.

### 3.3. PBC Tip Offset FEA Simulation

**Figure 17:**
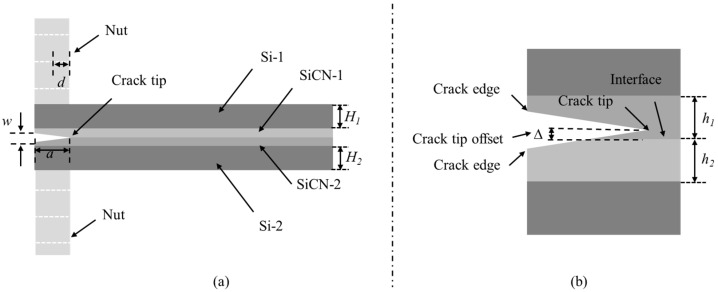
The PBC-DCB testing structure with crack tip offset. (**a**) Section view of PBC-DCB structure; the post-bending crack (PBC) has a parallel offset; (**b**) magnification of PBC, the crack tip is not coinciding with bonding interface, and the offset is ∆.

Theoretically, the “thickness” of a hybrid bonding interface is infinitesimally small. However, the high-resolution transmission microscope revealed that the interface has a thickness of 10 nm at most. The PBC crack is generated by precision laser cutting, while the laser or ion beam has an estimated inaccuracy of ~5 μm in alignment regarding the interface. Such misalignment may lead to crack propagation away from the interface (Figure 17). To evaluate the impact of such a tendency, an FEA simulation is performed.

**Figure 18:**
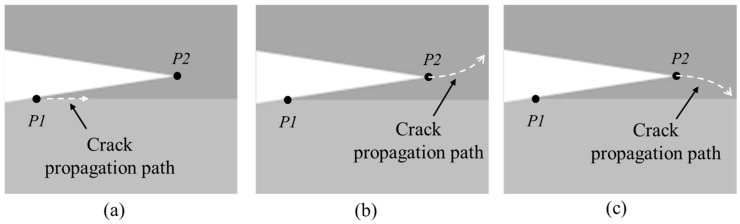
Crack tip offset and crack propagation path. (**a**) Crack growth from P1; (**b**) crack growth from P2 propagates upward; (**c**) crack growth from P2 propagates downward.

Whether the misalignment would divert the crack away from the interface depends on the geometry of the post-bonding crack regarding the whole specimen. From this aspect, determining the locations of the fracture initiation point and propagation path are the critical issues in the analysis, as shown in Figure 18.

Based on fracture mechanics theory [17], the fracture initiation point can be simplified into two possible points: P1 and P2 (Figure 18). If the crack initiation point is P1 and propagates along the bonding interface, the result is consistent with typical DCB tests. If initiation point is P2 and propagates upward, it may cause the fracture of the Si substrates. If the initiation point is P2 and propagates downward, the crack could propagate back to the bonding interface and then continue along the interface, which ends up being the same as that from P1.

(9) $$G=\frac{\partial U}{\partial A}=\frac{P^{2}}{2\omega }\cdot \frac{dC}{da}$$

This extends to nonlinear structures using the following equation:

(10) $$G=G\left(P,\delta ,a,Y\right)$$

where

*P* is the additional load;
δ is the displacement;
*C* is the compliance, C=P/δ;
*a* is the crack length;
Y is the factor of geometry.

According to Equation (10), the energy release rates of P1 and P2 are expressed as follows:

(11) $$G_{1}=G_{T1}\left(P_{1},\delta_{1},a_{1},Y_{1}\right)$$

(12) $$G_{2}=G_{T2}\left(P_{2},\delta_{2},a_{2},Y_{2}\right)$$

The fracture criterion is expressed as follows:

(13) $$f=\frac{G_{1}}{G_{2}}$$

If f>1, the crack will propagate from P2;
If f<1, the crack will propagate from P1.

**Figure 19:**
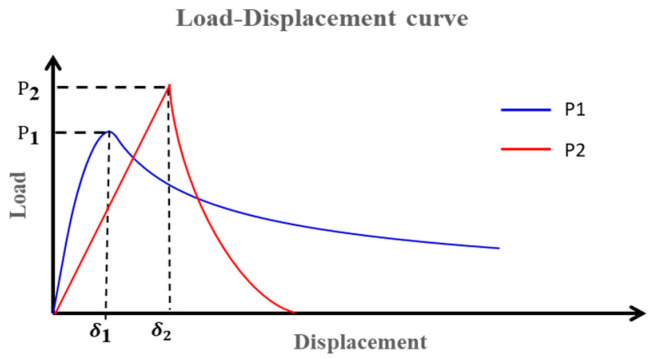
Load–displacement curves for crack propagation from P1 or P2 with the crack tip offset.

When applying the load to the cantilever beams with a displacement [10], cracks are assumed to propagate from the location of P1 or P2. The corresponding load–displacement curve can be obtained from different initiation points using FEA simulation. As shown in Figure 19, if $G_{1}<G_{2}$ and f<1, the crack propagation initiation point should be P1.

**Figure 20:**
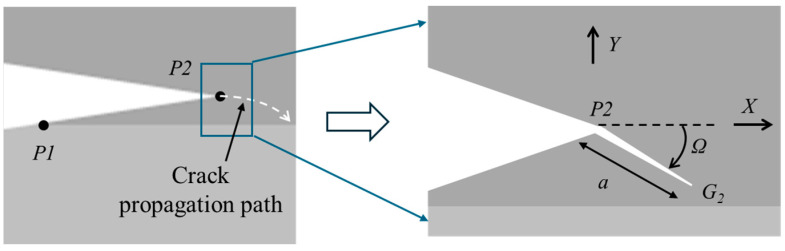
Kinking of a putative flaw out from P2 with the crack tip offset.

If $G_{1}>G_{2}$ and f>1, the crack propagation initiation point should be P2, as shown in Figure 20. According to kinking theory [18], the energy release rate is

(14) $$G_{kink}=G_{2}\cdot g(\varphi ,\Omega )$$

(15) $$\varphi ={tan}^{-1}K_{2}/K_{1}$$

where K_1_ and K_2_ are stress intensity factors of mode I and II.

According to (12)–(14), the kink angle Ω can be expressed as

(16) $$\Omega =\psi (P,\delta ,a,K_{i},Y)$$

(17) Y=Y(∆)

where ∆ is the crack tip offset geometry.

Accurate solutions can be obtained for a certain definite geometry, but it is often challenging for complex conditions or structures. The irregularity caused by crack tip offset makes it difficult to directly solve the stress intensity factors at the possible crack initiation points P1 and P2. In this part, the finite element method is used to numerically solve the crack propagation problems in a PBC-DCB structure with a misaligned crack tip.

**Figure 21:**
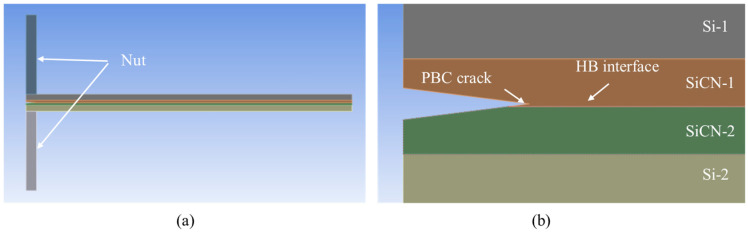
FEA of the PBC-DCB structure with crack tip offset. (**a**) Overview of whole structure; (**b**) magnification of crack tip offset.

A DCB 3D geometric structure with a triangular wedge pre-crack is illustrated in Figure 21, and the crack tip has an offset from the SiCN-SiCN bonding interface.

**Figure 22:**
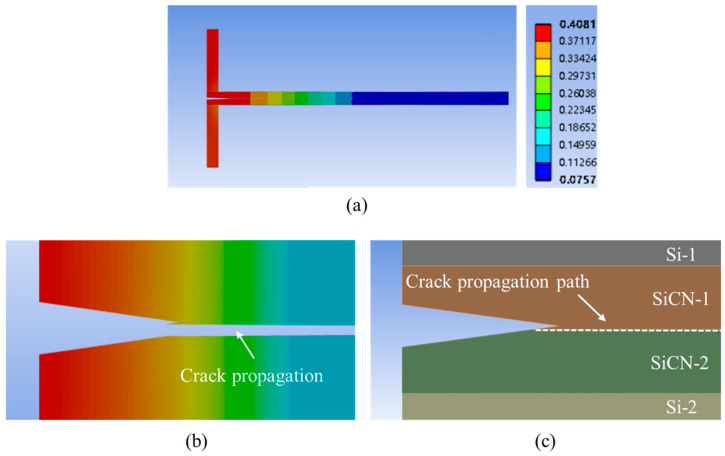
FEA simulation result of crack propagation with a crack tip offset of 2.0 μm. (**a**) Overview of the deformation for the whole model. (**b**) The deformation and crack propagation after simulation. (**c**) Schematic of the crack tip propagation path.

Based on the above principles, assuming the crack tip offset is 2.0 μm and the SiCN-SiCN bonding strength is 3.53 J/m^2^, the simulated crack propagation is shown in Figure 22.

From the simulation result, when the offset is very small, the possible crack propagation of P1 and P2 is almost the same, which means that the structural asymmetry caused by the pre-crack offset is relatively small. In this situation, the pre-crack still propagates along the bonding interface and DCB testing can be used for bonding strength testing.

**Figure 23:**
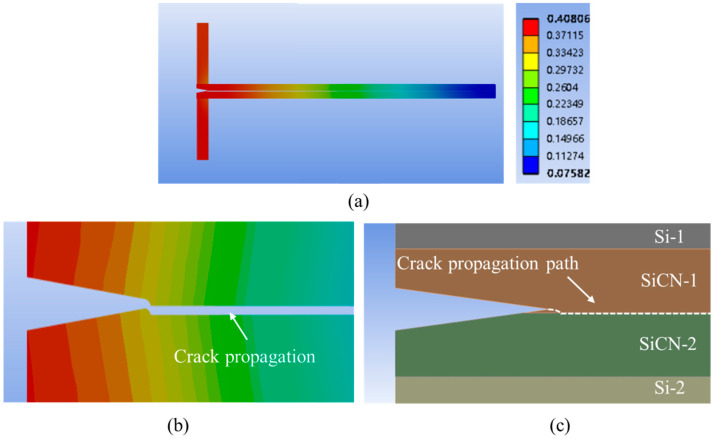
FEA simulation result of crack propagation with a crack tip offset of 10.0 μm. (**a**) Overview of the deformation for the whole model. (**b**) The deformation and crack propagation after simulation. (**c**) Schematic of the crack tip propagation path.

When the offset is increased to 10.0 μm, the crack propagation morphology is shown in Figure 23.

It can be found that when the offset is 10 μm, P2 is the initiation point of the crack propagation, and the crack extends towards the bonding interface. So, the crack propagation path is from P2 and rapidly extends to the bonding interface, then it continues to growth along the interface.

**Figure 24:**
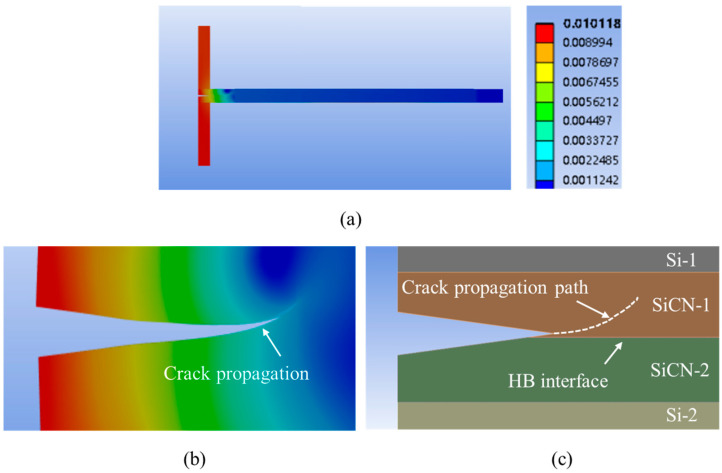
The FEA simulation result of crack propagation with a crack tip offset of 20.0 μm. (**a**) Overview of the deformation for the whole model. (**b**) The deformation and crack propagation after simulation. (**c**) Schematic of the crack tip propagation path.

When the pre-crack offset increased to 20.0 μm, the crack propagation morphology is shown in Figure 24. It is observed that the crack initiates at point P2 and subsequently deviates from the bonding interface, indicating that the crack propagation will proceed to the beam. So, in this case, the DCB testing is not suitable for bonding strength testing.

**Figure 25:**
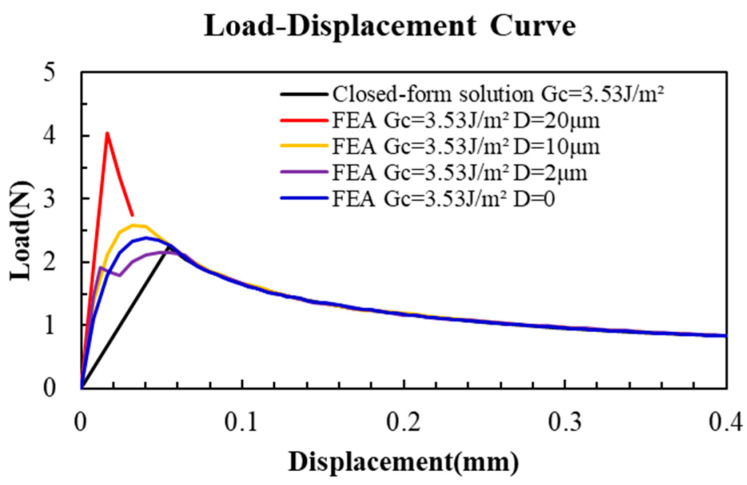
Finite element model simulation results for load–displacement curves with different values of crack tip offset.

All the load–displacement curves obtained by the different offset situations are plotted in Figure 25.

Based on the FAE result, the offset D can affect the direction and angle Ω of crack propagation from P1 or P2, as shown in Equation (18). The critical offset distance D_c_ is between 10 and 20 μm. When the PBC crack offset is small (D < D_c_), the PBC-DCB testing method is still available and reliable, which has been validated by the FAE simulation result. Considering the issue of PBC accuracy, the offset distance is suggested to be less than 10 μm.

(18) $$\Omega =\psi (P,∆,a,K_{i},D)$$

## 4. Conclusions

To solve the problem of accurately evaluating wafer-to-wafer hybrid bonding strength, this research first proposes an improved PBC-DCB method. Then this method is applied to test the interfacial bonding strength of SiCN-SiCN activated by different conditions. Referring to the PBC-DCB testing data, two FEA fracture models (VCCT and CZM) are discussed for the HB interface crack propagation simulation.

(1) For the PBC-DCB method, it employs post-preparation to generate the crack after hybrid bonding using a laser. This post-preparation can reduce the impact on the bonding interface and sample preparation. Furthermore, the nut placement also has been changed to decrease the half-length, which can improve testing accuracy. Then, this PBC-DCB method is applied to test the interfacial bonding strength of SiCN-SiCN activated by O_2_ or N_2_. The test data show that the bonding strengths are 3.53 J/m^2^ and 2.93 J/m^2^ with the deviation less than 3.84% and 1.84%. Based on the testing data, the influencing factors affecting the bonding strength calculation are discussed.
(2) To describe the crack propagation process in the PBC-DCB tests, finite element analysis models (including VCCT and CZM) are used. The results indicate that VCCT and CZM bilinear models are not suitable for this testing process. These models all assume that the load and displacement change linearly before and after crack propagation, which is inconsistent with the actual testing situation. Finally, a modified viscoelastic structure of exponential interface delamination model is introduced to accurately simulate the crack propagation behavior.
(3) For the PBC-DCB model with the crack tip offset issue, this optimized FEA model is applied to evaluate the PBC-DCB model with different crack tip offset values. The simulation results indicate that when the PBC tip offset is small, the crack will still propagate along the bonding interface during testing, so the PBC-DCB method is still available and reliable.

In the subsequent phase, our research will prioritize the extension of this optimized model to other dielectric materials (e.g., SiO_2_ and PI), while advancing the mechanical strength evaluation of Cu/SiO_2_ or Cu/SiCN hybrid bonding structures.

## Figures and Tables

**Table 1:** PBC-DCB parameters of SiCN-SiCN bonding.

Parameter	Symbol (Unit)	Value
Young’s modulus of Si	*E_s_* (GPa)	168
Poisson’s ratio of Si	*v_s_*	0.064
Young’s modulus of SiCN	*E_c_* (GPa)	208
Poisson’s ratio of SiCN	*v_c_*	0.22
The thickness of Si-1	*H*_1_ (mm)	0.75
The thickness of Si-2	*H*_2_ (mm)	0.75
The thickness of SiCN-1	*h*_1_ (mm)	0.3
The thickness of SiCN-2	*h*_2_ (mm)	0.3
The width of wafer	*w* (mm)	4.92
Half-length of nut	*d* (mm)	0.65

**Table 2:** The typical parameters of CZM bilinear model for interface delamination [16].

Parameter	Symbol	Group A	Group B	Group C
Maximum normal traction	$T_{max}$ (MPa)	0.1	0.05	0.01
Normal displacement jump at completion of debonding	$\delta_{f}$ (mm)	0.0586	0.1172	0.586

**Table 3:** CZM exponential model for interface delamination.

Parameter	Symbol (Unit)	Group A	Group B	Group C
Maximum Normal Traction	$\sigma_{max}$ (MPa)	0.1	0.05	0.01
Normal Separation Across the Interface	$\bar{\delta_{n}}$ (mm)	0.0586	0.1172	0.586
Equivalence factor	M	*e*	*e*	*e*
Bonding strength	$\emptyset_{n}$ (J/m^2^)	2.93	2.93	2.93

**Table 4:** Optimized CZM exponential model for interface delamination.

Parameter	Symbol (Unit)	Group A	Group D
Maximum Normal Traction	$\sigma_{max}$ (MPa)	0.1	0.1
Normal Separation Across the Interface	$\bar{\delta_{n}}$ (mm)	0.0586	0.075
Equivalence Factor	m	e	3.91
Bonding Strength	J/m^2^	2.93	2.93

## Data Availability

The original contributions presented in this study are included in the article. Further inquiries can be directed to the corresponding author.
